# Statin-induced metabolic reprogramming in head and neck cancer: a biomarker for targeting monocarboxylate transporters

**DOI:** 10.1038/s41598-018-35103-1

**Published:** 2018-11-14

**Authors:** Manal Mehibel, Fernando Ortiz-Martinez, Nadine Voelxen, Amy Boyers, Amy Chadwick, Brian A. Telfer, Wolfgang Mueller-Klieser, Catharine M. West, Susan E. Critchlow, Kaye J. Williams, Ian J. Stratford

**Affiliations:** 1 Division of Pharmacy and Optometry, School of Health Sciences, University of Manchester, Manchester, UK; 2 Institute of Pathophysiology, University Medical Centre of the Johannes Gutenberg University Mainz, Mainz, Germany; 3 Faculty of Biology, Division of Molecular & Clinical Cancer Sciences, Medicine and Health, University of Manchester, Manchester, UK; 4 Translational Radiation Biology, University of Manchester, The Christie NHS Foundation Trust, Manchester Academic Health Science Centre, Manchester, United Kingdom; 5 iMED Oncology, AstraZeneca, Cambridge, UK; 6 CRUK-EPSRC Cancer Imaging Centre in Cambridge and Manchester, Cambridge, UK

## Abstract

Prognosis of HPV negative head and neck squamous cell carcinoma (HNSCC) patients remains poor despite surgical and medical advances and inadequacy of predictive and prognostic biomarkers in this type of cancer highlights one of the challenges to successful therapy. Statins, widely used for the treatment of hyperlipidaemia, have been shown to possess anti-tumour effects which were partly attributed to their ability to interfere with metabolic pathways essential in the survival of cancer cells. Here, we have investigated the effect of statins on the metabolic modulation of HNSCC cancers with a vision to predict a personalised anticancer therapy. Although, treatment of tumour-bearing mice with simvastatin did not affect tumour growth, pre-treatment for 2 weeks prior to tumour injection, inhibited tumour growth resulting in strongly increased survival. This was associated with increased expression of the monocarboxylate transporter 1 (MCT1) and a significant reduction in tumour lactate content, suggesting a possible reliance of these tumours on oxidative phosphorylation for survival. Since MCT1 is responsible for the uptake of mitochondrial fuels into the cells, we reasoned that inhibiting it would be beneficial. Interestingly, combination of simvastatin with AZD3965 (MCT1 inhibitor) led to further tumour growth delay as compared to monotherapies, without signs of toxicity. In clinical biopsies, prediagnostic statin therapy was associated with a significantly higher MCT1 expression and was not of prognostic value following conventional chemo-radiotherapy. These findings provide a rationale to investigate the clinical effectiveness of MCT1 inhibition in patients with HNSCC who have been taking lipophilic statins prior to diagnosis.

## Introduction

Statins are structural analogues of mevalonate and act via inhibition of the 3-hydroxymethyl-3-glutaryl (HMG)-coenzyme A (CoA) reductase to competitively block the conversion of HMG-CoA to mevalonate, a precursor in the synthesis of cholesterol^1^. Statin therapy is recommended for the treatment of hyperlipidemia as well as the primary and secondary prevention of cardiovascular diseases^2^. Studies have shown that statins also possess powerful pleiotropic effects independent of their lipid-lowering effects in a wide range of pathological disorders such as inflammatory diseases, Alzheimer’s disease, Parkinson’s disease and multiple sclerosis^1,3^. Recently, there are emerging interests in the use of statins as anticancer agents based on preclinical evidence of their antiproliferative, proapoptotic and tumouricidal properties^4,5^ and their use was found to be associated with a significant reduction in cancer-related deaths in 13 distinct cancer types^6^. Inhibition of 3-hydroxy-3-methylglutaryl CoA reductase by statins leads to reduced levels of mevalonate and its downstream products, many of which play critical roles in cellular functions such as membrane integrity, cell signaling, cell cycle progression^7,8^ and mitochondrial damage^9^. In addition, glucose uptake was shown to be impaired in cancer cells exposed to statins^10^. Glucose metabolism in cancer is largely regulated by monocarboxylate transporters (MCTs). MCTs are a family of transmembrane proteins that mediate the transport of monocarboxylates such as pyruvate and lactate, with MCT1 and MCT4 being the main ones implicated in cancer^11^.

The aim of this project was to investigate potential statin-induced metabolic modulation in head and neck squamous cell carcinoma (HNSCC) with particular emphasis on the expression of MCT1 and MCT4 and whether statin use could be of predictive value in designing a personalised anti-cancer therapy. This is particularly important since, with the exception of HPV infection, there is a lack of predictive and prognostic biomarkers in this type of cancer^12^. It is a heterogeneous disease comprising a group of tumours that include the oral cavity, nasal cavity, paranasal sinuses, pharynx, larynx and salivary glands and is the sixth most common cancer in the world, accounting for more than 550,000 new cases each year^13^. Overall survival rates remain poor despite surgical and medical advances, with less than 50% of patients surviving at 5 years and hence locoregional control remains the main focus of disease management^14,15^. Therefore, there is an urgent need to develop more effective therapies for this deadly disease.

## Results

### Sensitivity of HNSCC cancer cell lines to statins

**Figure 1:**
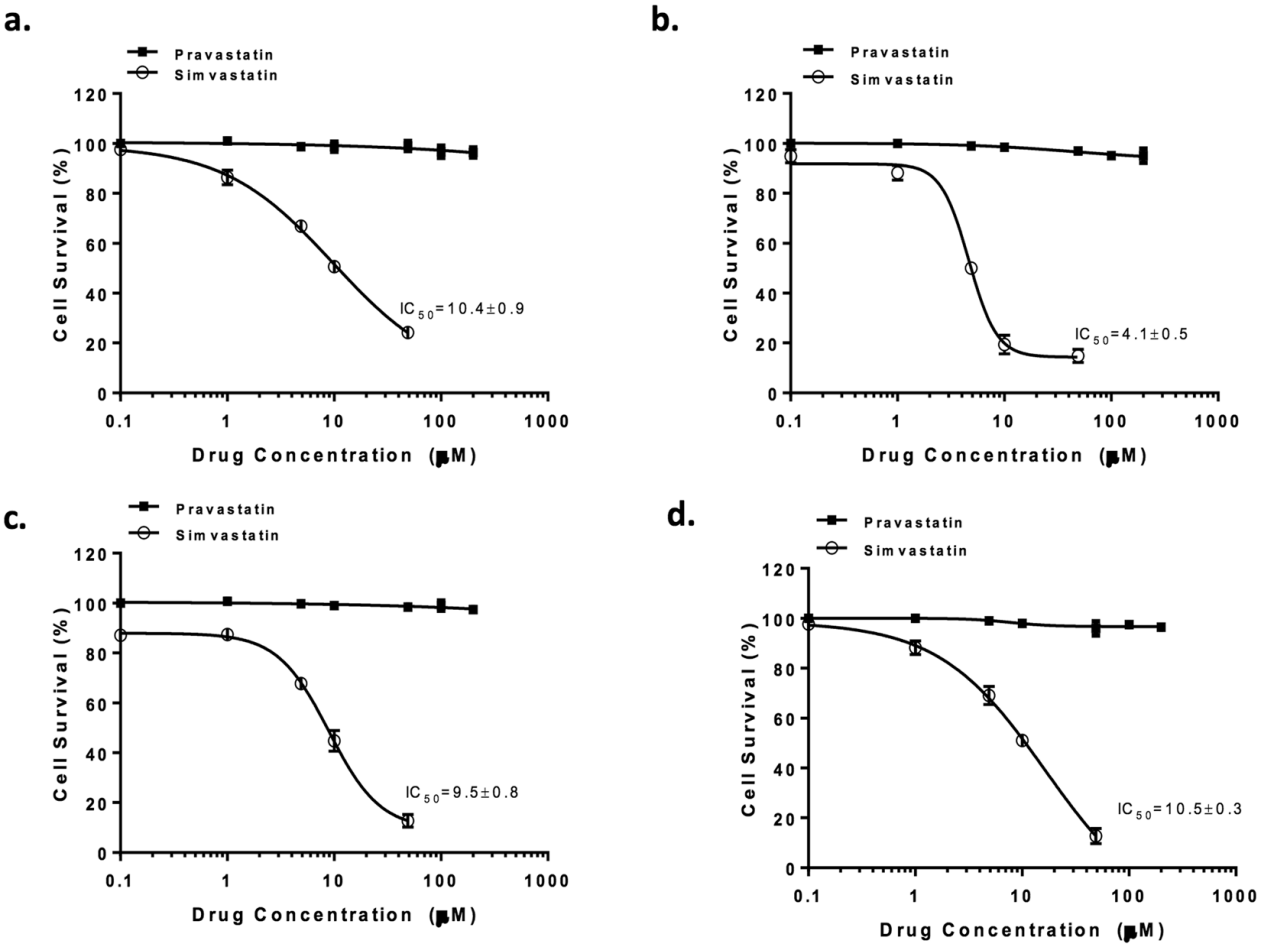
Cellular toxicity of statins is dependent on their lipophilicity. Four HNSCC cell lines were treated with either simvastatin or pravastatin for 96 hours and effect was determined by SRB assay. Data is presented as the percentage of cells unaffected by the drug relative to untreated control. (**a**) FaDu; (**b**) CaL-27; (**c**) Detroit 562; (**d**) UMSCC47. All graphs represent mean of at least three independent experiments carried out in triplicate.

A panel of four HPV negative HNSCC cancer cell lines (Supplementary Table 1) were employed to assess efficacy of statins. The cells were exposed to either simvastatin (lipophilic) or pravastatin (hydrophilic) for 96 hours. It is worth noting that simvastatin is clinically administered as the prodrug lactone which is activated *in vivo* chemically or enzymatically by esterases or paraoxonases^16^. *In vitro*, the prodrug was activated to its active acid form by hydrolysis prior to treatment. Simvastatin effectively inhibited cell proliferation in a dose-dependent manner in all the cell lines tested with IC_50_ values ranging between 4–10 μM, while pravastatin showed no cellular toxicity up to a concentration of 200 μM (Fig. 1). This is in agreement with previous findings in a panel of breast cancer cells, where it was suggested that lack of toxicity is due to pravastatin’s hydrophilic nature which prevents it from efficiently permeating cell membranes^17^. Although the active forms of simvastatin and pravastatin are similar in structure, pravastatin is approximately 100 times less lipophilic than simvastatin. In fact, pravastatin is mainly metabolised by hydroxylation and this results in more hydrophilic metabolites. On the other hand, simvastatin acid, the main metabolite of simvastatin is still lipophilic. The greater hydrophilicity of pravastatin has been suggested as a reason for its selectivity with respect to inhibition of cholesterol synthesis and lower permeation into nonhepatic tissues^18^.

HPV positivity does not seem to play a role in the sensitivity of the cells to simvastatin since both UMSCC47 (HPV positive), FaDu and Detroit 562 (HPV negative) have similar IC_50_ values.

Based on these findings, the lipophilic simvastatin was taken forward for further investigation.

### Chronic simvastatin treatment results in metabolic reprogramming

**Figure 2:**
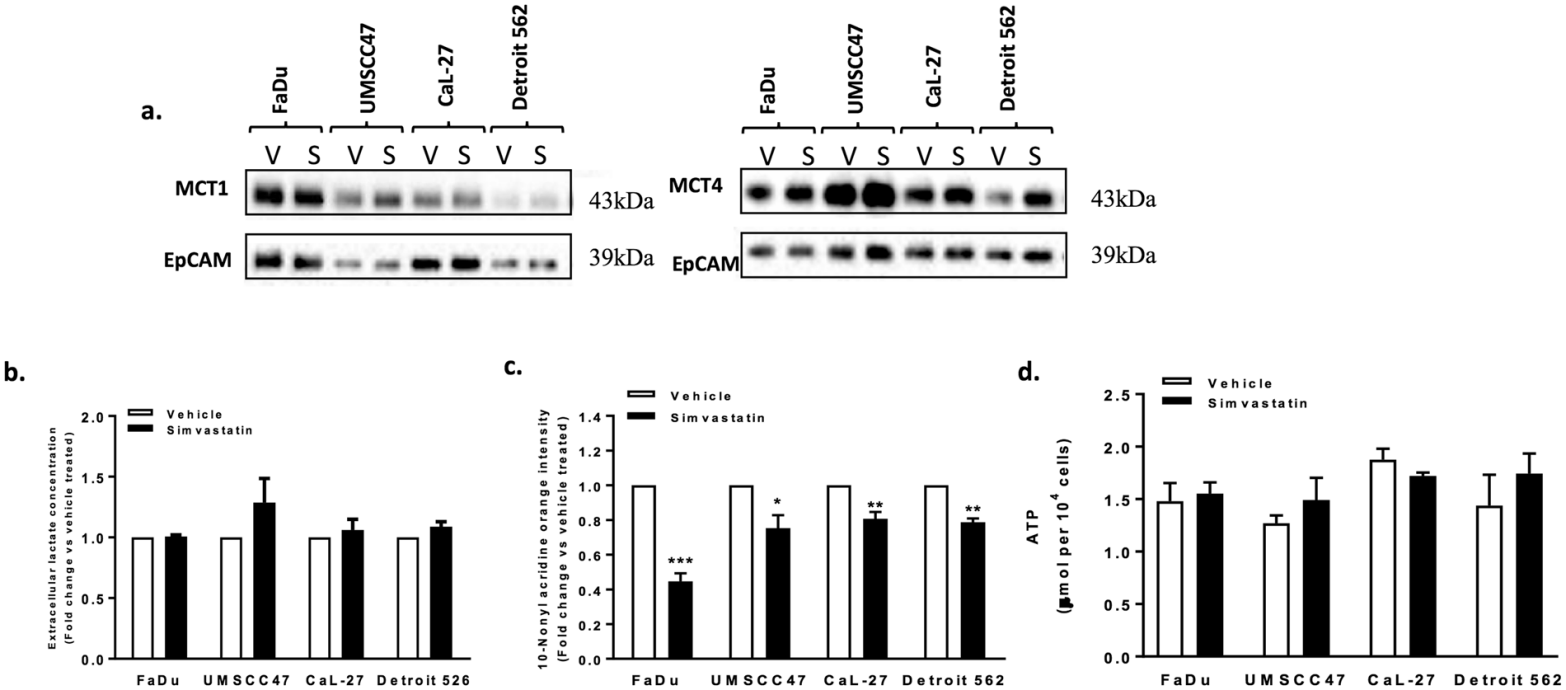
Simvastatin treatment is associated with metabolic changes. (**a**) HNSCC cell lines were treated with simvastatin or vehicle for 96 hours. Cell surface MCTs were isolated by biotinylation and assayed by western blotting. EpCAM (plasma membrane marker) was used as a loading control. (**b**) Extracellular lactate was measured and expressed relative to vehicle-treated cells. (**c**) Simvastatin affects mitochondrial mass, cells were stained with 10-nonyl acridine orange (10-NAO) and signal was quantified using flow cytometry. (**d**) ATP measurements in vehicle and simvastatin-treated cells. Bars represent mean of at least three independent experiments. *P < 0.05, **P < 0.01 & ***P < 0.001, *t*-test.

The four cell lines were then exposed to simvastatin for 96 hours at sub IC_50_ concentrations (CaL-27 cells were treated with 2 μM and the other cells with 5 μM). Cell surface proteins were isolated through biotinylation to ensure that only expression of membrane-bound MCTs is assessed. This is necessary because only membrane-bound MCTs are functional and therefore are actively involved in regulating glycolysis^19^. We found that chronic treatment with statins led to changes in expression of membrane-bound MCTs, with an increase in MCT4 expression levels observed in all four cell lines, although changes in MCT1 expression were less dramatic (Fig. 2a). Surprisingly, statin treatment was not associated with changes in extracellular lactate levels (Fig. 2b).

10-NAO is an orange dye associated with mitochondrial cardiolipin and is frequently used to evaluate total mitochondrial mass^20^. Simvastatin therapy markedly reduced mitochondrial mass in the four cancer cells tested with the most profound effect observed in FaDu cells (Fig. 2c). Interestingly, simvastatin therapy did not result in changes of ATP levels in the four cell lines tested suggesting that cell death is not caused by ATP depletion (Fig. 2d).

### Combining simvastatin treatment with inhibition of MCT function ***in vitro***

**Figure 3:**
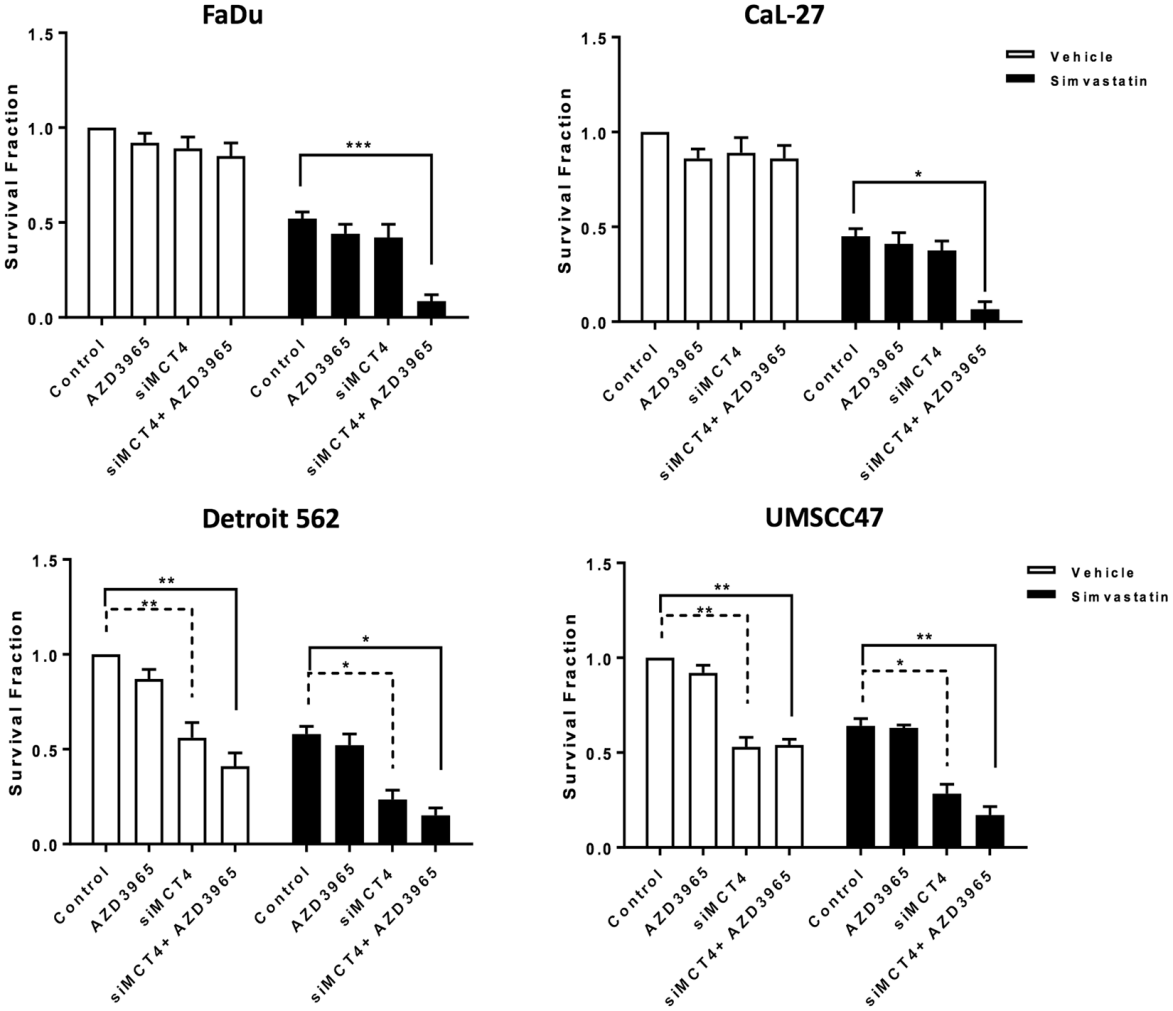
Cell survival of HNSCC panel after MCT inhibition with and without pre-treatment with simvastatin, as determined by clonogenic survival assay. Cells were pre-exposed to vehicle or simvastatin for 96 hours, then subjected to treatment with AZD3965 (1 mM) for 72 hours and/or siMCT4. Bars represent mean of at least three independent experiments carried out in duplicate. *P < 0.05, **P < 0.01, ***P < 0.001, one-way ANOVA test.

Based on the finding that chronic treatment with simvastatin is associated with changes in expression levels of MCTs, we hypothesised that combining statin treatment with MCT inhibition could be a useful therapeutic strategy. MCT1 inhibition was achieved pharmacologically using the AZD3965 drug at a concentration of 1 mM for 72 hours. AZD3965 is an orally bioavailable compound currently in clinical trials for treatment of cancer (NCT01791595)^21^. Since there are no available specific pharmacologic inhibitors of MCT4, genetic knockdown was employed (Supplementary Fig. 1 shows efficiency of MCT4 knockdown). The cells were initially pre-treated with simvastatin for 96 hours which was then followed by treatment with siMCT4 and/or AZD3965 for 72 hours. Cellular toxicity was assessed by clonogenic assay and the results are presented in Fig. 3.

For vehicle treated FaDu and CaL-27 cells, treatment with siMCT4 and/or AZD3965 did not have a significant effect on cellular toxicity. Treatment with simvastatin alone resulted in a significant reduction in survival. Exposing these two cells to simvastatin followed by either siMCT4 or AZD3965 alone did not enhance toxicity, possibly due to the fact that each transporter compensates for the inhibition of the other one^22^. Interestingly, combining simvastatin treatment with inhibition of both transporters resulted in a significant enhancement in toxicity.

Vehicle treated Detroit 562 and UMSCC47 cells showed enhanced sensitivity to MCT4 but not MCT1 inhibition, suggesting that MCT4 is the most crucial to their cellular function. This is consistent with the previous finding by Baek and colleagues that MCT4 inhibition only affects viability in MCT4-addicted cells^23^. Inhibition of both transporters did not result in further toxicity in these cells and a similar trend was observed when the cells were pre-treated with simvastatin. Therefore, in these cells pre-exposure to simvastatin does not sensitise them to inhibition of MCT function.

Investigations of metabolic pathways *in vitro* are limited by the fact that the milieu does not mimic the tumour microenvironment. This is of particular importance since the finding of metabolic symbiosis phenomenon and the involvement of both MCT1 and MCT4^24^. It was therefore imperative to translate our findings into relevant *in vivo* models and for this purpose the two most sensitive cell lines FaDu and CaL-27 were used.

### *In vivo* toxicity of simvastatin

**Figure 4:**
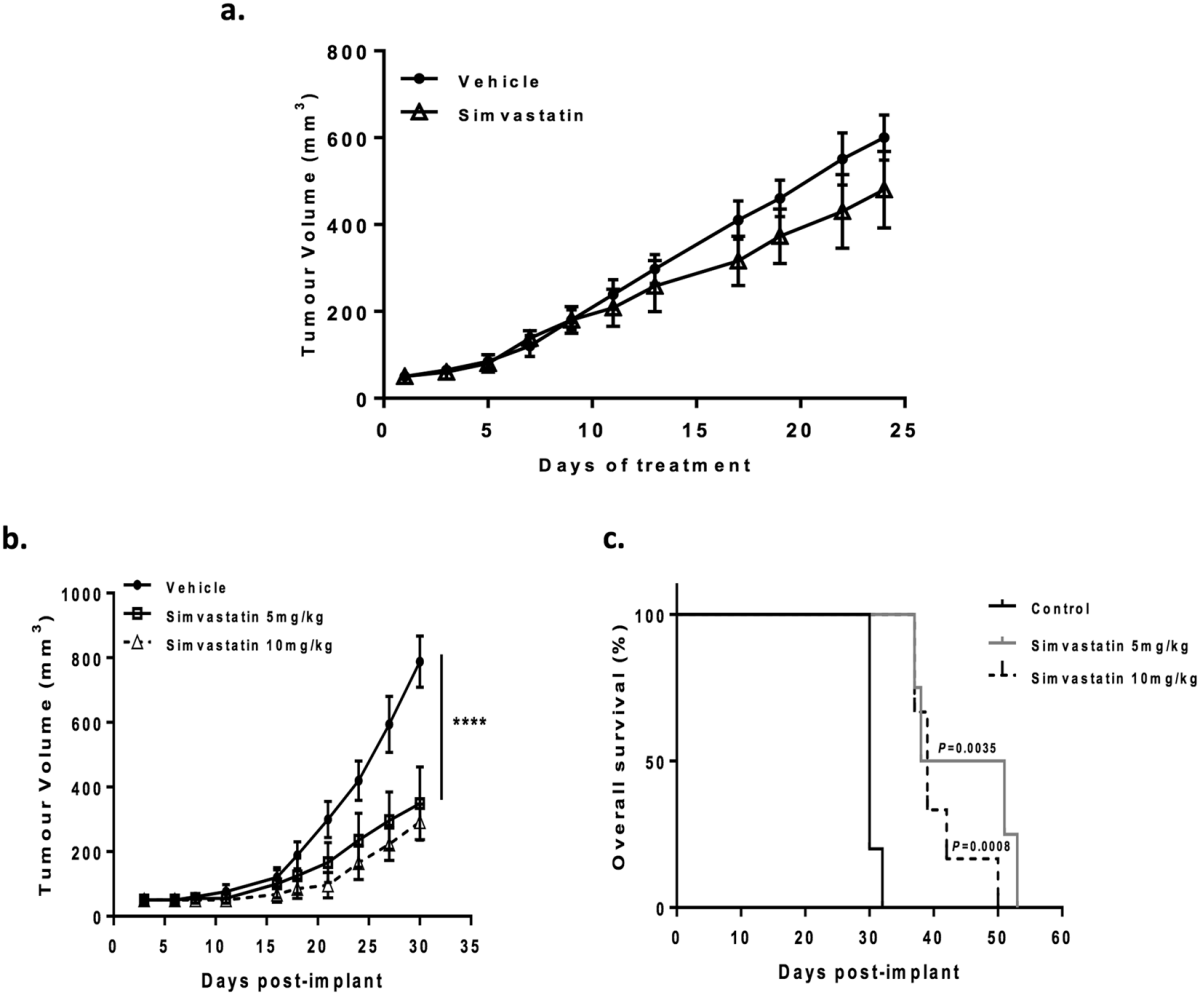
Effect of simvastatin therapy *in vivo*. (**a**) Simvastatin has no effect on the growth of established FaDu xenografts. When tumour volumes reached 50 mm^3^, mice were randomized into two groups which were treated with vehicle or simvastatin (10 mg/kg) daily by oral gavage. Tumour volumes were determined every 2–3 days. Total number of animals used 14. (**b**) Simvastatin inhibits the growth FaDu tumours. Mice were dosed once daily with simvastatin (5 mg/kg or 10 mg/kg) or vehicle for 2 weeks prior to s.c. injection of tumour cells, dosing continued until tumour volumes reached 800 mm^3^, ****P < 0.0001, one-way ANOVA test. (**c**) Kaplan-Meier survival curves and log-rank *P* values. Total number of animals used 21.

To investigate whether simvastatin has chemotherapeutic effects *in vivo*, mice with established FaDu xenografts were given simvastatin at oral daily doses of 10 mg/kg when tumour volumes reached 50 mm^3^. This dose would equate to ∼40 mg/day for an adult weighing 60 kg, based on mouse-to-human equivalence calculations^25^, which is within the recommended dose range of statins in the clinic^26^. Unfortunately, in this case simvastatin did not have any effects on tumour growth (Fig. 4a).

In order to mimic a clinical scenario where patients who present with a tumour would have been taking simvastatin for months to years, mice were given oral daily doses of simvastatin two weeks prior to injection of tumour cells. Dosing continued throughout the study with two clinically relevant doses 5 mg/kg and 10 mg/kg, which are pre-standardised and well-tolerated doses for *in vivo* studies^4^. Interestingly, both doses were efficient at significantly reducing FaDu tumour growth (Fig. 4b; P < 0.0001; one-way ANOVA) and improving overall survival (Fig. 4c). However, a less dramatic albeit significant growth delay was observed in CaL-27 tumours (Supplementary Fig. 2). Simvastatin at these oral doses was well-tolerated as evident in the absence of changes in body weight and the general condition of the mice (Supplementary Figs 3 and 4).

### Simvastatin therapy causes a metabolic switch in tumours

**Figure 5:**
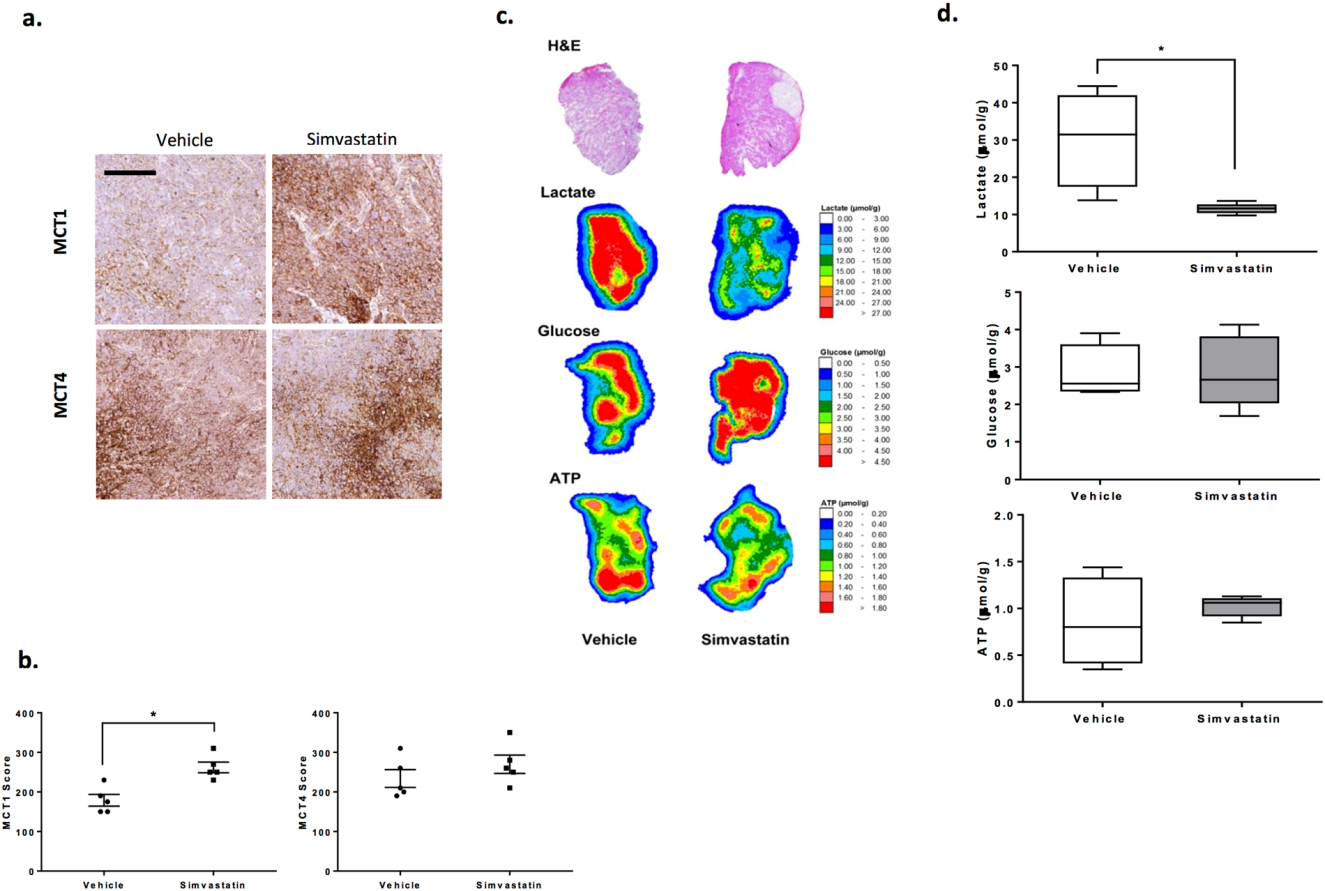
Simvastatin therapy is associated with upregulation of MCT1 expression and a metabolic shift *in vivo*. (**a**) Representative images of MCT1 and MCT4 immunohistochemical staining in FaDu tumours, scale bar represents 200 μm. (**b**) Semiquantitative analysis of MCT1 and MCT4 expression in tumours treated with 10 mg/kg simvastatin or vehicle. (**c**) Measurements of ATP, lactate and glucose levels in FaDu tumor xenografts by imBI. hematoxylin and eosin (H&E) staining and color-coded distributions of lactate, glucose and ATP in sequential cryosections from representative tumors. The concentration values were color-coded, with each color corresponding to a defined concentration range in μmol/g. (**d**) Concentrations of metabolites in FaDu tumors. ATP, Lactate and glucose values were calculated from simvastatin-treated or vehicle-treated tumors. The data correspond to the mean of at least four independent experiments in which one mouse was used per experimental condition. *P < 0.05, *t*-test.

Tumours at sizes of 150 mm^3^ were collected for immunohistochemical analyses of monocarboxylate transporters. Simvastatin therapy was associated with a significant increase in membrane bound MCT1, while no significant differences in MCT4 staining patterns were observed between vehicle and simvastatin-treated tumours (Fig. 5a,b).

To better characterise metabolic changes following therapy *in vivo*, we measured metabolites (ATP, lactate and glucose) in these tumours using the induced metabolic bioluminescence imaging (imBl) technique. Representative images of the analysis are presented in Fig. 5c, where each color represents a pre-defined concentration range for the measured metabolites.

Measurements of lactate levels disclosed drastically lower lactate concentrations in tumours from simvastatin-treated mice compared with control samples. For FaDu xenografts, lactate content was 30.3 ± 6.4 μmol/g in vehicle-treated tumours versus 11.5 ± 0.6 μmol/g in simvastatin-treated tumours (P = 0.01). Therapy did not result in changes in ATP or glucose levels (Fig. 5d). ATP concentrations generally reflect the viability of the tested tissue sections and it is noteworthy that only samples of an ATP level of ≥0.2 μmol/g are classified as viable and were included in the study^27^. In the CaL-27 model, tumours treated with simvastatin had a lactate level of 10.2 ± 0.9 μmol/g compared to 17.9 ± 3.2 μmol/g in vehicle treated tumours (P = 0.01) and again no changes were observed in the measurements of ATP or glucose (Supplementary Fig. 5). These metabolic changes could suggest that simvastatin-treated tumours rely on oxidative phosphorylation and resort to the upregulation of MCT1 expression as a compensatory mechanism for survival, consistent with the role of MCT1 an importer of mitochondrial fuels^24^. Theoretically, this could result in enhancement of sensitivity to inhibitors of MCT1 function.

### Tumour xenografts treated with simvastatin are sensitive to MCT1 inhibition

**Figure 6:**
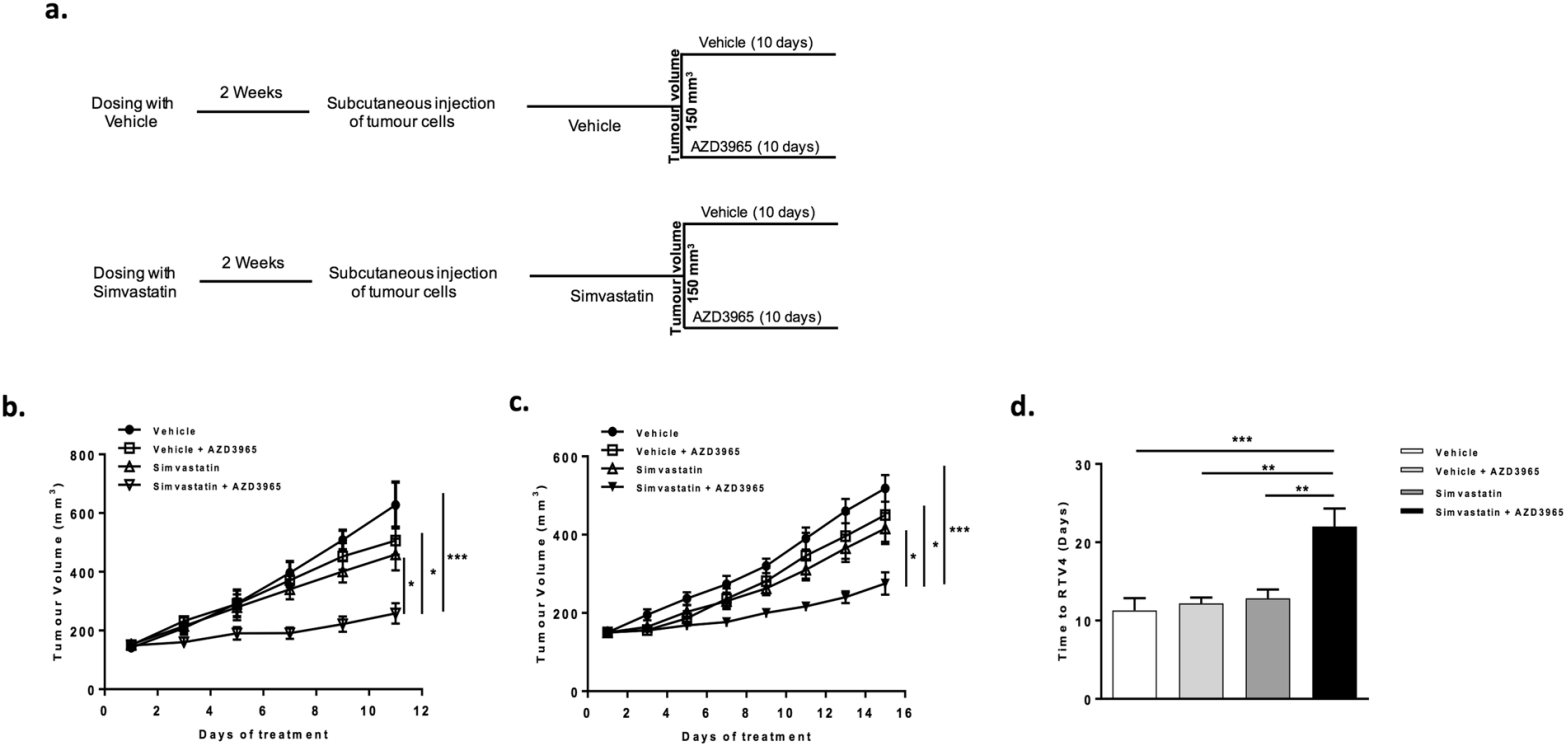
Combination therapy of simvastatin and AZD3965 efficiently inhibits growth of human xenograft tumours. (**a**) Layout of the experiment: mice were dosed once daily with simvastatin (10 mg/kg) or vehicle for 2 weeks prior to tumour cells injection, dosing continued throughout the experiment. When tumour volume reached 150 mm^3^, each group was further randomised to receive vehicle or AZD3965 (100 mg/kg) *BID* for 10 days. (**b**) *In vivo* growth curves of FaDu xenografts grown in CD-1 nude mice, showing tumour volume after start of AZD3965 treatment. Total number of animals used 28. (**c**) *In vivo* growth curves for CaL-27 xenografts grown in SCID mice, showing tumour volume after start of AZD3965 treatment. Total number of animals used 24. (**d**) Time (days) taken for FaDu xenografts to reach four times the initial volume (time to RTV4). *P < 0.05 & ***P < 0.001, one-way ANOVA test.

Mice receiving vehicle or simvastatin at daily oral dose of 10 mg/kg for 2 weeks prior to tumour injection, were given vehicle or the MCT1 inhibitor AZD3965 for 10 days starting when the tumours reached a size of 150 mm^3^ as illustrated in Fig. 6a. AZD3965 was administered by oral gavage at a dose of 100 mg/kg twice daily which is a well-tolerated dose for *in vivo* studies^22,28^. AZD3965 monotherapy did not result in any toxicity which is possibly due to the fact that both xenograft tumours express high levels of MCT4 (Supplementary Fig. 6) which is a well-known resistance factor to MCT1 inhibition^22^. Interestingly, combination therapy of AZD3965 and simvastatin resulted in greater inhibitory effect on tumour growth as compared to simvastatin or AZD3965 monotherapies in both xenograft tumours (Fig. 6b,c). Analysis of the time taken for individual FaDu tumours to grow from 150 to 600 mm^3^ showed that this was 11 days for tumours in mice treated with vehicle, 12 days for those given AZD3965 alone or simvastatin alone compared with 22 days for combination therapy of simvastatin and AZD3965 (Fig. 6d). All treatments were well-tolerated as there were no significant changes in body weight (Supplementary Fig. 5).

### Prediagnostic statin use is associated with upregulation of MCT1 expression

Our preclinical data suggest that statin therapy is associated with an upregulation of MCT1 expression and enhanced sensitivity to MCT1 inhibition with AZD3965. To investigate the clinical significance of this finding in patients with HNSCC, we analysed a cohort of 119 clinical biopsies taken at diagnosis prior to therapy. Supplementary Table 2 shows the descriptive statistics for the study patient population and representative images of immunohistochemically staining of MCTs are depicted in Supplementary Fig. 7A. Each stained section was scored for MCT expression independently by two researchers using predetermined scoring criteria (see Materials and Methods and Supplementary Fig. 7B). Validation of MCT scoring was achieved by independent scoring by a pathologist.

Patients were also stratified according to their pre-diagnostic statin use. Exclusion criteria from the study were the use of hydrophilic statins (pravastatin and rosuvastatin) since we and others have confirmed that they don’t exert antitumour effects^17,29^. The other exclusion criterion was metformin use because of its well-known metabolic effects in cancer^30^.

Patients were taking simvastatin or atorvastatin for at least three months prior to diagnosis with HNSCC. The Subcohort of patients who were on these statins prediagnostically had significantly higher tumour MCT1 expression than non-statin users (P = 0.02), 42% of statin users had high MCT1 expression versus only 16% in non-users and MCT4 expression was not significantly different between the two groups (P = 0.19) confirming that statin use is associated with an upregulation of only MCT1 expression in HNSCC tumours (Table 1).

**Table 1:** Prediagnostic statin use is associated with high MCT1 expression in HNSCC clinical biopsies.

	High MCT1	Low MCT1	*P* value	High MCT4	Low MCT4	*P* value
Control (*n* = 100)	16%	84%	**0**.02*	31%	69%	0.19
Statin users (*n* = 19)	42%	58%		47%	53%	

Percentage of patients with high or low MCT expression in non-statin users (control) and statin users. *P* value calculated using Fisher’s exact test.

**Figure 7:**
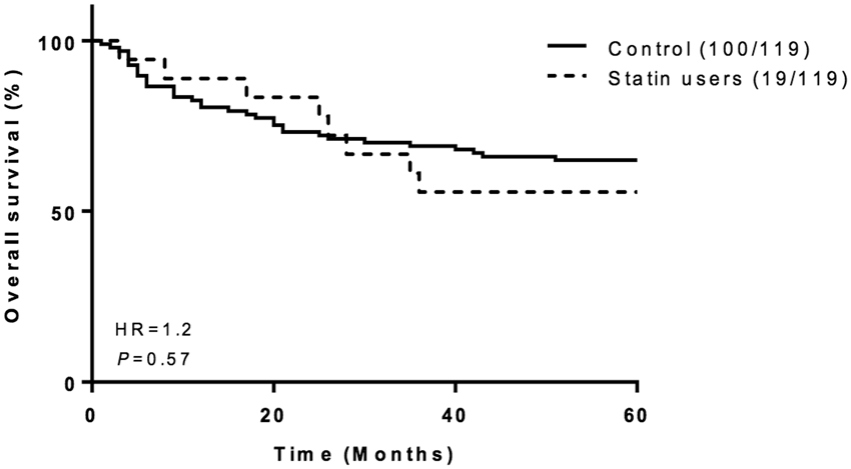
Pre-diagnostic statin use does not affect clinical outcome. Kaplan-Meier survival curves showing the relationship between overall survival and pre-diagnostic statin use in 119 HNSCC patients. The *P* value (log rank test) and hazard ratio (HR) for each comparison are shown.

Kaplan-Meier survival analysis demonstrates that prediagnostic statin use did not affect patient survival and was therefore of no prognostic significance in this cohort (P = 0.57; hazard ratio (HR) = 1.2; Fig. 7).

## Discussion

The data on the link between statins and cancer is currently equivocal due to inconsistent findings reported by several studies. Some stated that the use of statins was associated with a significant reduction in overall cancer incidence as well as cancer-related deaths^6,31^, while others found no association between statin use and mortality from or incidence of cancer^32^. There are even reports of a positive association between long-term statin use and incidence of invasive lobular carcinomas and ductal carcinomas and the increased risk was limited to patients who used lipophilic statins for at least 10 years^33^. Ahern and colleagues reported that the use of lipophilic simvastatin was related to a decrease in the rate of breast cancer recurrence in women with invasive breast cancer while such association was not observed with hydrophilic statins^34^. Our *in vitro* toxicity data suggest that lipophilic but not hydrophilic statins can exert anti-cancer effects and this is consistent with previous findings in both breast and gynecological cancers^7,17^. It is well-known that the more lipophilic statins efficiently permeate extrahepatic tissues while the more hydrophilic compounds are more confined to the liver^18^ leading to the conclusion that lipophilic statins have the greatest potential in exerting pleiotropic effects. It is possible that previous epidemiological data on the relationship between statins and cancer have been confounded by the lack of distinction between lipophilic and hydrophilic statins.

Several studies have addressed the antitumour effects of statins *in vivo*^4,17,29^ but not in the context of glucose metabolism. In our study, we show that simvastatin antagonises tumour metabolic reprogramming, an important hallmark of cancer. Here, we show for the first time that simvastatin caused a stable modification in the metabolic phenotype of head and neck tumours as demonstrated by disturbances in glycolysis-associated markers.

Induced metabolic bioluminescence imaging is a very useful technique for metabolic profiling of tumours as well as tracking changes in the glycolytic pathways associated with therapy^35^. The technique has already been applied to clinical samples from patients with cervical carcinomas^36^, squamous cell carcinomas of the head and neck^37^, rectal adenocarcinomas^38^ and more recently, advanced ovarian carcinomas^27^. Our metabolic imaging results clearly show that simvastatin drastically reduced lactate levels in xenografts. Lactate, a key regulator of the glycolytic phenotype, is a potent oncometabolite that accelerates tumour development, facilitates metastasis and supports resistance to radiotherapy^37,39^. Another key finding of the study is the demonstration that simvastatin therapy was associated with overexpression of MCT1 in both clinical biopsies and xenograft tumours. Interestingly, it has previously been reported that high expression of MCT1 correlates with high oxidative and low glycolytic status in some tumour cells, consistent with its role as an importer of lactate and ketone bodies into the cells for the purpose of energy production^24,40^. Therefore, we can conclude that the tumours treated with simvastatin are characterised by a poorly glycolytic phenotype compared to their control counterparts. The associated overexpression of MCT1 is an indicator of dependency of statin treated tumours on this transporter for efficient metabolism as a compensatory survival mechanism, suggesting that blocking this compensatory pathway could be of therapeutic benefit.

Deregulation of cellular metabolism is an important hallmark of cancer^41^ and several novel strategies have been employed to interfere with the efficient metabolic pathways, including inhibition of monocarboxylate transporters^22,24^. AZD3965, an MCT1 inhibitor currently in clinical trials, has been shown to reduce tumour growth through inhibition of lactate release^22,28^. It has also been suggested that MCT1 inhibition prevents MCT1-mediated lactate uptake into aerobic cells where it is generally converted to pyruvate for further energy production, thereby forcing aerobic cells to use glucose for metabolism which decreases its availability for the hypoxic cells that are reliant on it for their survival^24^. In the present study, head and neck tumour xenografts treated with simvastatin showed enhanced sensitivity to MCT1 inhibition with AZD3965 as compared to vehicle treated tumours, suggesting that simvastatin could be a useful predictive indicator for the success this therapy.

High MCT1 expression is associated with poor clinical outcomes in bladder, and small cell lung cancers^22,42^. In head and neck cancers, it was MCT4 expression that had prognostic significance^43^ and was also associated with poor prognosis in prostate and colorectal cancers^44,45^. It is important to highlight that in our study, simvastatin therapy was not associated with changes in MCT4 expression in both tumour xenografts and clinical biopsies, which is consistent with lack of detrimental effect on overall patient survival. Although, a recent study showed that statin use at the time of diagnosis of HPV negative head and neck cancer was associated with improved survival^46^.

In recent years, there has been a shift towards personalised, patient-tailored treatment in cancer therapy and predictive biomarkers are potentially the most useful for clinical decision-making. Our data strongly suggest that prediagnostic statin use warrants further clinical investigation as a predictive biomarker for targeting metabolic pathways in tumours through inhibition of MCT1 function. This strategy is of particular relevance to HPV negative HNSCC since success of therapy has been hindered by the lack of clinically useful prognostic and predictive biomarkers in this type of cancer^12^. Although, our study has focused on the advantage of prediagnostic statin therapy, we believe that there may still be benefits to combination therapies with statins initiated after diagnosis.

## Materials and Methods

### Reagents

For *in vitro* studies, simvastatin (Enzo Life Sciences) was dissolved in ethanol and NaOH, neutralized to pH 7.2 for activation as a 20 mM stock solution as previously described^4^. Pravastatin (Sigma) was dissolved in water as a 20 mM stock solution. AZD3965 (AstraZeneca) was prepared as a 1 mM stock solution in DMSO. Stock solutions of drugs were stored as single use aliquots at −20 °C. All other reagents were of high analytical grades and obtained from Sigma unless otherwise stated.

### Cell culture

FaDu, Detroit 562, CaL-27 (American Type Culture Collection, Manassas, VA) and UMSCC47 (kind donation from Prof. Chris Boshoff, UCL Cancer Institute, London) were maintained in a humidified atmosphere at 37 °C and 5% CO_2_.

FaDu cells were maintained in EMEM and the other cell lines in RPMI 1640. Culture medium was supplemented with 10% fetal calf serum (Life technologies) and 2 mmol/L glutamine. Cell lines were routinely screened for the presence of Mycoplasma (Venor^™^ GeM Mycoplasma Detection Kit) and authenticated using the Powerplex 16HS system (Promega) throughout the study.

### Concentration response assays

Cells were seeded at appropriate seeding densities into 96-well plates, allowed to attach for 3 hours before treatment with different concentrations of simvastatin or pravastatin for 96 hours. Plates were then stained with Sulforhodamine B (SRB) and processed as previously described^47^ to assess cellular toxicity.

### Lactate Measurement

Lactate concentration was determined by colorimetric assay (Trinity Biotech) according the manufacturer’s instructions. Briefly, the concentration of lactate in cell-free medium was detected by spectrophotometry at 450 nm using a standard curve of known lactate concentrations.

### Measurement of ATP levels and mitochondrial mass

ATP levels were measured using the ATPlite™ Luminescence Assay System (Perkin Elmer) according to manufacturer’s instructions. Cells were seeded and exposed to 100 µl of medium with and without simvastatin for 96 hours. At endpoint, lysis solution was added to stop the reaction followed by substrate solution and luminescence was measured.

Mitochondrial mass was measured by staining live cells with 10-Nonyl acridine orange dye (Molecular probes, Invitrogen). Cells were grown to 70% confluency in a 6-well dish. The dye was diluted in RPMI media (final concentration: NAO = 20 nM) and exposed to the cells for 30 minutes at 37 °C. Cells were then washed with PBS, collected by scraping and analysed using LSRFortessa flow cytometer (BD Biosciences).

### Biotinylation of cell surface MCTs/Cell surface protein isolation

For the isolation and collection of surface proteins, the Pierce® Cell Surface Protein Isolation Kit (Thermo Scientific) was used as recommended by the manufacturer’s protocol. Details are reported under Supplementary Methods.

### Immunoblot Assays

Protein from whole cell lysates was quantified using the Pierce BCA Protein Assay^48^ with bovine serum albumin as a protein standard. Equal amounts of proteins were separated by SDS–PAGE and transferred onto a nitrocellulose membrane. After blocking with 5% milk for 1 hour, membranes were probed with primary antibodies against MCT4 (sc50329, Santa Cruz biotechnology), MCT1 (sc50324, Santa Cruz biotechnology), EpCAM (ab32392, Abcam), cyclophilin A (ab41684, Abcam) and β-Actin (A1978, Sigma) overnight at 4 °C. Proteins were detected by horseradish-peroxidase linked secondary antibodies and chemiluminescent signal detected using ECL Plus (Bio-Rad). Since the proteins are of close molecular weights, gels were stripped in Restore Plus western blot stripping buffer for subsequent re-blotting.

### Clonogenic assays

Cells were seeded in 6 well plates and allowed to attach for 3 hours before treatment with the appropriate concentrations of simvastatin. After 96 hours, cells were transfected with siMCT4 and/or treated with 1 mM AZD3965 or vehicle for 72 hours in the presence of simvastatin. Medium was then changed and cells allowed to form colonies. At the end of the experiment, the medium was removed and the colonies were fixed in 70% ethanol and stained with methylene blue to facilitate counting of colonies (≥50 cells). Clonogenic survival was calculated for each condition after correcting for plating efficiency. Survival fraction was calculated by dividing survival of each condition by survival of vehicle-only treated cells.

### Gene silencing by RNA interference

Transfections of siRNA duplexes targeting MCT4 or a nontargeting control (NT) (ON-TARGETplus SMARTpool, GE Healthcare) at a final concentration of 25 nM, were performed in Optimem (Invitrogen), using Dharmafect 1 reagent (GE Healthcare).

### *In vivo* Efficacy Studies

Mice were maintained in a sterile environment, housed in individually vented caging systems in a 12-hour light/12-hour dark environment and maintained at uniform temperature and humidity. FaDu xenografts were established by subcutaneous injection of 1 × 10^6^ cells in 0.1 ml serum-free medium into the mid-dorsal flank of 8- to 12-week-old female CD-1 nude mice (Charles River, Wilmington, MA, USA). CaL-27 xenografts were established by injection of 5 × 10^6^ in 0.1 ml serum- free medium/Matrigel mix into the mid-dorsal flank of 8- to 14-week-old female SCID mice (Charles River).

When FaDu tumours reached an average size of 50 mm^3^, mice were randomised and divided into 2 groups, according to treatments administered by oral gavage. The first group of mice was treated daily with 0.1 ml of vehicle (0.5% methylcellulose), the second group of mice was treated daily with 0.1 ml of drug (5 mg/kg or 10 mg/kg simvastatin in 0.5% methylcellulose). For subsequent experiments, oral daily dosing with vehicle or simvastatin (10 mg/kg) started two weeks prior to cell inoculation. Treatment was continued and tumour-bearing mice were randomly assigned into further treatment groups of eight once a tumour volume of 150 mm^3^ had been achieved. The group of mice receiving vehicle was randomised into two groups and treated with 100 mg/kg BID AZD3965 in 0.5% hydroxypropyl methyl cellulose, 0.1% tween 80 or vehicle only by oral gavage for 10 days. The group of mice receiving simvastatin was also randomised into two groups and treated with 100 mg/kg BID AZD3965 in 0.5% hydroxypropyl methyl cellulose, 0.1% tween 80, or simvastatin only by oral gavage for 10 days. Simvastatin and vehicle dosing continued until the end of the experiment. Tumour size was measured twice to three times a week using calipers. At endpoint, animals were sacrificed following the standard protocols and tumours for analysis were dissected from the neighbouring connective tissue. Specimens of tumours were snap frozen in liquid nitrogen and stored at −80C for imBl analysis of metabolites. The remaining tumour tissues of each sample were fixed in 10% formalin and embedded in paraffin for histological analysis. All animal study protocols were approved by the University of Manchester Animal Welfare and Ethics Review Body and the Home Office and designed in accordance with the Scientific Procedures Act (1986) and the 2010 guidelines for the welfare and use of animals in cancer research^49^. Animal numbers of each group were calculated by power analysis and animals were grouped randomly for each experiment.

### Patients’ samples and data collection

Formalin-fixed, paraffin-embedded primary head and neck tumour samples and clinical data were obtained from 119 consenting adult patients presenting to hospitals in the North West region of England between 2000 and 2010. Clinical biopsies were collected, processed and analysed in accordance with the relevant guidelines and regulations. For tissue microarray construction, tumour-rich regions (guided by histological review) from each case were sampled using 1-mm cores. All the archival paraffin-embedded tumour samples were coded with no patient identifiers. The study was approved by Tameside & Glossop Local Research Ethics Committee and informed consent was obtained for sample collection and analysis.

Information on the use of statins (type, dose, start date and duration) was derived from patient medical notes available at the Christie Foundation Trust (Manchester, UK) and confirmation of such details was obtained from GPs. All data was collected according to local ethical approvals.

### Immunohistochemistry (IHC)

Formalin-fixed paraffin-embedded 4 μm sections were prepared from the TMA or tumour xenografts. For MCT1 and MCT4 staining, sections were dewaxed, rehydrated, antigen retrieval was carried out in citrate buffer (pH 6), for 12 minutes at 98 ^o^C in an EZ retriever microwave and endogenous peroxidase blocked with 3% H_2_O_2_ for 15 minutes at room temperature. Slides were blocked with 10% casein solution for 30 minutes. The primary antibodies used were rabbit anti-MCT4 (H-90, Santa Cruz biotechnology) used at 1:500 dilution, and rabbit anti-MCT1 (AstraZeneca in-house antibody) used at 1:250 dilution. Secondary antibody reagent HRP-conjugated anti-rabbit Envision reagent was applied for 30 minutes and staining was visualized by incubation in DAB solution for 5 minutes. Slides were counterstained with hematoxylin, dehydrated, and mounted. As an isotype control, a rabbit IgG fraction was used in place of the primary antibodies. Positive control sections were employed in the study. Human placenta was used as a positive control for MCT4, and human colon was used as a positive control for MCT1. MCT1 expression was analysed on consecutive tumour sections to those utilised in the analysis of MCT4. Analysis of IHC staining is reported under Supplementary Methods.

### Induced metabolic bioluminescence imaging (imBl)

Snap-frozen tumours were cut into serial cryosections for metabolic measurements and structural hematoxylin and eosin staining. For quantitative measurement of ATP, lactate and glucose, the method of metabolic imaging with induced bioluminescence (imBI) was applied, as previously described^35,36,50^. Further details of imBI are reported under Supplementary Methods.

### Statistical analysis

All bar and line graphs represent mean ± Standard Error (SEM). Statistics were carried out using Fisher’s exact test or Student’s t-tests to compare differences between two groups. One-way analysis of variance was employed for comparison if there were more than two groups. Differences were considered statistically significant at P < 0.05. For xenograft tissue experiments, images are representative of cohorts of at least three mice. Kaplan–Meier analysis was used to assess overall survival (OS) rates over time, and log-rank (Mantel-Cox) test performed to determine whether observed differences between groups were statistically significant. Data were analysed using GraphPad Prism® v6.0 software.

## Electronic supplementary material

Supplementary information

## Data Availability

All data generated or analysed during this study are included in this published article (and its Supplementary Information files).

## References

[1] Liao JK, Laufs U (2005). Pleiotropic effects of statins. Annual review of pharmacology and toxicology.

[2] Lipinski MJ, Abbate A, Fuster V, Vetrovec GW (2007). Drug insight: statins for nonischemic heart failure–evidence and potential mechanisms. Nature clinical practice. Cardiovascular medicine.

[3] Mihos CG, Santana O (2011). Pleiotropic effects of the HMG-CoA reductase inhibitors. International journal of general medicine.

[4] Cho SJ (2008). Simvastatin induces apoptosis in human colon cancer cells and in tumor xenografts, and attenuates colitis-associated colon cancer in mice. International journal of cancer. Journal international du cancer.

[5] Kotamraju S, Williams CL, Kalyanaraman B (2007). Statin-induced breast cancer cell death: role of inducible nitric oxide and arginase-dependent pathways. Cancer research.

[6] Nielsen SF, Nordestgaard BG, Bojesen SE (2012). Statin use and reduced cancer-related mortality. The New England journal of medicine.

[7] Kato S (2010). Lipophilic but not hydrophilic statins selectively induce cell death in gynaecological cancers expressing high levels of HMGCoA reductase. Journal of cellular and molecular medicine.

[8] Goldstein JL, Brown MS (1990). Regulation of the mevalonate pathway. Nature.

[9] Cafforio P, Dammacco F, Gernone A, Silvestris F (2005). Statins activate the mitochondrial pathway of apoptosis in human lymphoblasts and myeloma cells. Carcinogenesis.

[10] Malenda A (2012). Statins impair glucose uptake in tumor cells. Neoplasia.

[11] Halestrap AP, Wilson MC (2012). The monocarboxylate transporter family–role and regulation. IUBMB life.

[12] Thomas GR, Nadiminti H, Regalado J (2005). Molecular predictors of clinical outcome in patients with head and neck squamous cell carcinoma. International journal of experimental pathology.

[13] Jemal A (2011). Global cancer statistics. CA: a cancer journal for clinicians.

[14] Erkal HS, Mendenhall WM, Amdur RJ, Villaret DB, Stringer SP (2001). Synchronous and metachronous squamous cell carcinomas of the head and neck mucosal sites. Journal of clinical oncology: official journal of the American Society of Clinical Oncology.

[15] Snow GB, Annyas AA, van Slooten EA, Bartelink H, Hart AA (1982). Prognostic factors of neck node metastasis. Clinical otolaryngology and allied sciences.

[16] Duggan DE, Vickers S (1990). Physiological disposition of HMG-CoA-reductase inhibitors. Drug metabolism reviews.

[17] Campbell MJ (2006). Breast cancer growth prevention by statins. Cancer research.

[18] Serajuddin AT, Ranadive SA, Mahoney EM (1991). Relative lipophilicities, solubilities, and structure-pharmacological considerations of 3-hydroxy-3-methylglutaryl-coenzyme A (HMG-CoA) reductase inhibitors pravastatin, lovastatin, mevastatin, and simvastatin. Journal of pharmaceutical sciences.

[19] Halestrap AP (2012). The monocarboxylate transporter family–Structure and functional characterization. IUBMB life.

[20] Maftah A, Petit JM, Ratinaud MH, Julien R (1989). 10-N nonyl-acridine orange: a fluorescent probe which stains mitochondria independently of their energetic state. Biochemical and biophysical research communications.

[21] Cancer Research UK. A Phase I Trial of AZD3965 in Patients With Advanced Cancer. NIH Web site (2015) Available from: http://www.clinicaltrials.gov/ct2/show/NCT01791595.

[22] Polanski R (2014). Activity of the monocarboxylate transporter 1 inhibitor AZD3965 in small cell lung cancer. Clinical cancer research: an official journal of the American Association for Cancer Research.

[23] Baek G (2014). MCT4 defines a glycolytic subtype of pancreatic cancer with poor prognosis and unique metabolic dependencies. Cell reports.

[24] Sonveaux P (2008). Targeting lactate-fueled respiration selectively kills hypoxic tumor cells in mice. The Journal of clinical investigation.

[25] Reagan-Shaw S, Nihal M, Ahmad N (2008). Dose translation from animal to human studies revisited. FASEB journal: official publication of the Federation of American Societies for Experimental Biology.

[26] Stone NJ (2014). 2013 ACC/AHA guideline on the treatment of blood cholesterol to reduce atherosclerotic cardiovascular risk in adults: a report of the American College of Cardiology/American Heart Association Task Force on Practice Guidelines. Circulation.

[27] Battista MJ (2016). Feasibility of induced metabolic bioluminescence imaging in advanced ovarian cancer patients: first results of a pilot study. Journal of cancer research and clinical oncology.

[28] Bola BM (2014). Inhibition of monocarboxylate transporter-1 (MCT1) by AZD3965 enhances radiosensitivity by reducing lactate transport. Molecular cancer therapeutics.

[29] Coimbra M (2010). Liposomal pravastatin inhibits tumor growth by targeting cancer-related inflammation. Journal of controlled release: official journal of the Controlled Release Society.

[30] Pernicova I, Korbonits M (2014). Metformin–mode of action and clinical implications for diabetes and cancer. Nature reviews. Endocrinology.

[31] Graaf MR, Beiderbeck AB, Egberts AC, Richel DJ, Guchelaar HJ (2004). The risk of cancer in users of statins. Journal of clinical oncology: official journal of the American Society of Clinical Oncology.

[32] Li X, Wu XB, Chen Q (2014). Statin use is not associated with reduced risk of skin cancer: a meta-analysis. British journal of cancer.

[33] Strandberg TE (2004). Mortality and incidence of cancer during 10-year follow-up of the Scandinavian Simvastatin Survival Study (4S). Lancet.

[34] Ahern TP (2011). Statin prescriptions and breast cancer recurrence risk: a Danish nationwide prospective cohort study. Journal of the National Cancer Institute.

[35] Curtarello M (2015). VEGF-targeted therapy stably modulates the glycolytic phenotype of tumor cells. Cancer research.

[36] Walenta S (2000). High lactate levels predict likelihood of metastases, tumor recurrence, and restricted patient survival in human cervical cancers. Cancer research.

[37] Brizel DM (2001). Elevated tumor lactate concentrations predict for an increased risk of metastases in head-and-neck cancer. International journal of radiation oncology, biology, physics.

[38] Walenta S (2003). Metabolic classification of human rectal adenocarcinomas: a novel guideline for clinical oncologists?. Journal of cancer research and clinical oncology.

[39] Blatt Sebastian, Voelxen Nadine, Sagheb Keyvan, Pabst Andreas Max, Walenta Stefan, Schroeder Thies, Mueller-Klieser Wolfgang, Ziebart Thomas (2016). Lactate as a predictive marker for tumor recurrence in patients with head and neck squamous cell carcinoma (HNSCC) post radiation: a prospective study over 15 years. Clinical Oral Investigations.

[40] Kim Y, Choi JW, Lee JH, Kim YS (2015). Expression of lactate/H(+) symporters MCT1 and MCT4 and their chaperone CD147 predicts tumor progression in clear cell renal cell carcinoma: immunohistochemical and The Cancer Genome Atlas data analyses. Human pathology.

[41] Hanahan D, Weinberg RA (2011). Hallmarks of cancer: the next generation. Cell.

[42] Choi JW, Kim Y, Lee JH, Kim YS (2014). Prognostic significance of lactate/proton symporters MCT1, MCT4, and their chaperone CD147 expressions in urothelial carcinoma of the bladder. Urology.

[43] Curry JM (2013). Cancer metabolism, stemness and tumor recurrence: MCT1 and MCT4 are functional biomarkers of metabolic symbiosis in head and neck cancer. Cell cycle.

[44] Pertega-Gomes N (2011). Monocarboxylate transporter 4 (MCT4) and CD147 overexpression is associated with poor prognosis in prostate cancer. BMC cancer.

[45] Nakayama Y (2012). Prognostic significance of monocarboxylate transporter 4 expression in patients with colorectal cancer. Experimental and therapeutic medicine.

[46] Lebo N (2018). Effect of statin use on oncologic outcomes in head and neck squamous cell carcinoma. Head & Neck.

[47] Vichai V, Kirtikara K (2006). Sulforhodamine B colorimetric assay for cytotoxicity screening. Nature protocols.

[48] Smith PK (1985). Measurement of protein using bicinchoninic acid. Analytical biochemistry.

[49] Workman P (2010). Guidelines for the welfare and use of animals in cancer research. British journal of cancer.

[50] Mueller-Klieser W, Walenta S (1993). Geographical mapping of metabolites in biological tissue with quantitative bioluminescence and single photon imaging. The Histochemical journal.

