# Supplementary material for: Statin-induced metabolic reprogramming in head and neck cancer: a biomarker for targeting monocarboxylate transporters
Source: Sci Rep. 2018 Nov 14;8:16804. doi: 10.1038/s41598-018-35103-1 (PMC6235971; doi:10.1038/s41598-018-35103-1)
Supplement: Supplementary file 1 — Supplementary information [file 41598_2018_35103_MOESM1_ESM.pdf]

Statin-induced metabolic reprogramming in head and neck cancer: a biomarker for targeting monocarboxylate transporters

Manal Mehibel*, Fernando Ortiz-Martinez, Nadine Voelxen, Amy Boyers, Amy Chadwick, Brian A Telfer, Wolfgang Mueller-Klieser, Catharine ML West, Susan E Critchlow, Kaye J Williams, Ian J Stratford.

Supplementary methods and materials

Measurement of ATP levels and mitochondrial mass

ATP levels were measured using the ATPlite™ Luminescence Assay System (Perkin Elmer) according to manufacturer's instructions. Cells were seeded and exposed to 100 µl of medium with and without simvastatin for 96 hours. At endpoint, lysis solution was added to stop the reaction followed by substrate solution and luminescence was measured.

Mitochondrial mass was measured by staining live cells with 10-Nonyl acridine orange dye (Molecular probes, Invitrogen). Cells were grown to 70% confluency in a 6-well dish. The dye was diluted in RPMI media (final concentration: NAO = 20 nM) and exposed to the cells for 30 minutes at 37°C. Cells were then washed with PBS, collected by scraping and analysed using LSRFortessa flow cytometer (BD Biosciences).

Biotinylation of cell surface MCTs/ Cell surface protein isolation

Cells were washed with ice-cold PBS followed by incubation with 0.25 mg/mL Sulfo-NHS-SS-Biotin in 48 mL ice-cold PBS per flask on a rocking platform for 30 minutes at 4ºC. The biotinylation reaction was quenched by adding 500 µL of the provided Quenching Solution. Cells were harvested by gentle scraping, pelleted by centrifugation and lysed using the provided Lysis Buffer containing a protease inhibitor cocktail for 30 minutes on ice with intermittent vortexing. The lysates were then centrifuged and the clarified supernatant was used for purification of biotinylated proteins on NeutrAvidin Agarose. The captured surface proteins were eluted from the biotin-NeutrAvidin Agarose by incubation with dithiothreitol (DTT) in PBS containing 62.5 mM Tris-HCl for 1 h at room temperature. The eluted proteins, representing the cell surface proteins, were collected by column centrifugation at 1,000 x *g* for 2 minutes. For all cell lines, three biological replicates were obtained. Protein concentrations were quantified using the BCA protein Assay Kit (Pierce) and the lysates were stored at -20ºC until use.

Analysis of IHC staining

IHC staining was scored by two independent researchers and validated by a clinical pathologist blinded to all clinico-pathological variables. Protein expression was scored for membrane MCT1 and MCT4 as both proteins are functional at the plasma membrane. Areas of necrosis, stroma, normal epithelium and distinct edge effects were ignored.

Immunoreactivity was scored for both the intensity and the proportion of cells stained; intensity was given scores of 0-4 (0, no staining; 1, very weak staining; 2, weak staining; 3, moderate staining; 4, strong staining), as shown in **Supplementary Figure 7**. The two scores were multiplied to obtain the final score within the range of 0-400. MCT score ≥200 and <200 were described as high and low, respectively.

Induced metabolic bioluminescence imaging

Snap-frozen tumours were cut into serial cryosections for structural hematoxylin and eosin (H&E) staining and metabolic measurements. The spatial concentrations of ATP, glucose, and lactate in cryosections of tumours were obtained using the method of metabolic imaging with induced bioluminescence (imBI), as previously described (20, 21). For measurement,

metabolites are enzymatically linked to the light reaction of bioluminescence enzymes, leading to light emission with the intensity being proportional to the tissue content of each metabolite.

Light emission was induced in a temperature-stabilized reaction chamber, which was placed under a microscope (Axiophot, Zeiss, Oberkochen, Germany) connected to a 16bit CCD camera with an imaging photon counting system (iXon^{EM}+ DU-888, Andor Technology PLC, Belfast, Northern Ireland). Light intensities were calibrated using appropriate standards. Metabolite content was calculated in µmol/g tumour tissue and images were displayed in colors coding for tissue concentration of metabolites in units of µmol/g.

Computerised image analysis allowed for separate data assessment in selected histological areas of xenograft tumours. Five tumours of vehicle-treated and simvastatin-treated were analysed using three sections from each tumour. Pixel values were summarised for individual tumours into one distribution histogram. From this histogram, mean values(± SEM) and additional statistical parameters were calculated. All metabolite concentrations shown here were acquired exclusively from vital tumour regions.

Supplementary Data

Cell line	Pathology	HPV Status
FaDu	Squamous cell carcinoma of the hypopharynx	Negative
Detroit 562	Squamous cell carcinoma of the pharynx	Negative
CaL-27	Squamous cell carcinoma of the tongue	Negative
UM-SCC-47	Squamous cell carcinoma of lateral tongue	HPV-16 Positive

Supplementary Table 1: Pathology and HPV status of HNSCC panel used in the study

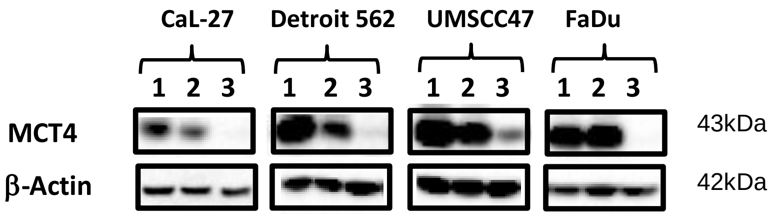

1. Untransfected
2. Non-targeting siRNA
3. siMCT4

Supplementary Figure 1: Efficiency of transient knockdown of MCT4 in the HNSCC panel. Cells were transfected with the non-targeting siRNA or siMCT4 for 24 hours. Proteins collected 96 hours post transfection and assayed by western blotting. β-Actin was used as a loading control. Blots are representatives of three independent experiments.
1: untransfected, 2: non-targeting siRNA, 3: siMCT4.

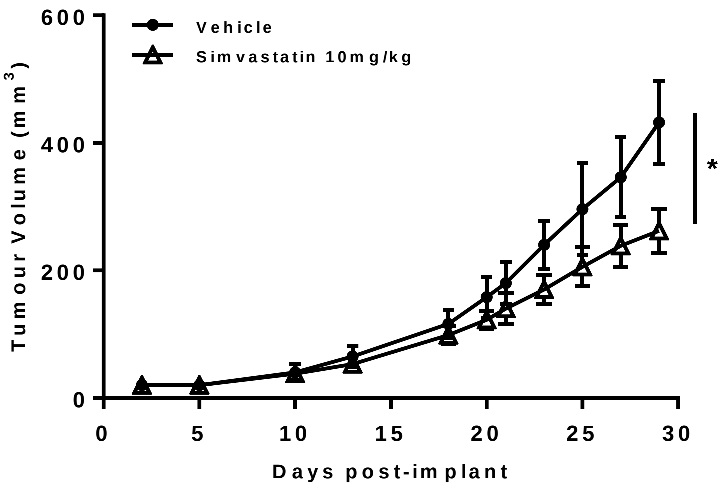

Supplementary Figure 2: Simvastatin inhibits growth of CaL-27 xenografts. Mice were dosed once daily with simvastatin (10mg/kg) or vehicle for 2 weeks prior to s.c. injection of tumour cells, dosing continued until end of experiment. Total number of animals used 12. **P* <0.05, *t*-test.

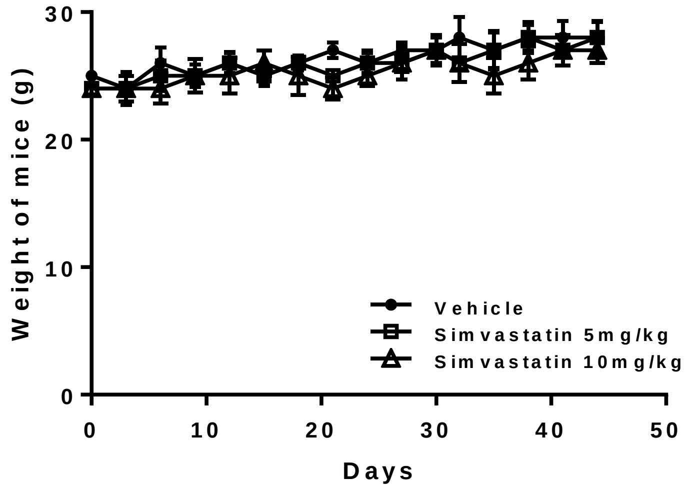

Supplementary Figure 3: Body weights of tumour-bearing mice were not affected by treatment with simvastatin. Athymic CD-1 Nude mice received s.c. injections of FaDu cells. Body weights were measured every 2–3 d during the time period of treatment with vehicle or simvastatin (5mg/kg or 10mg/kg).

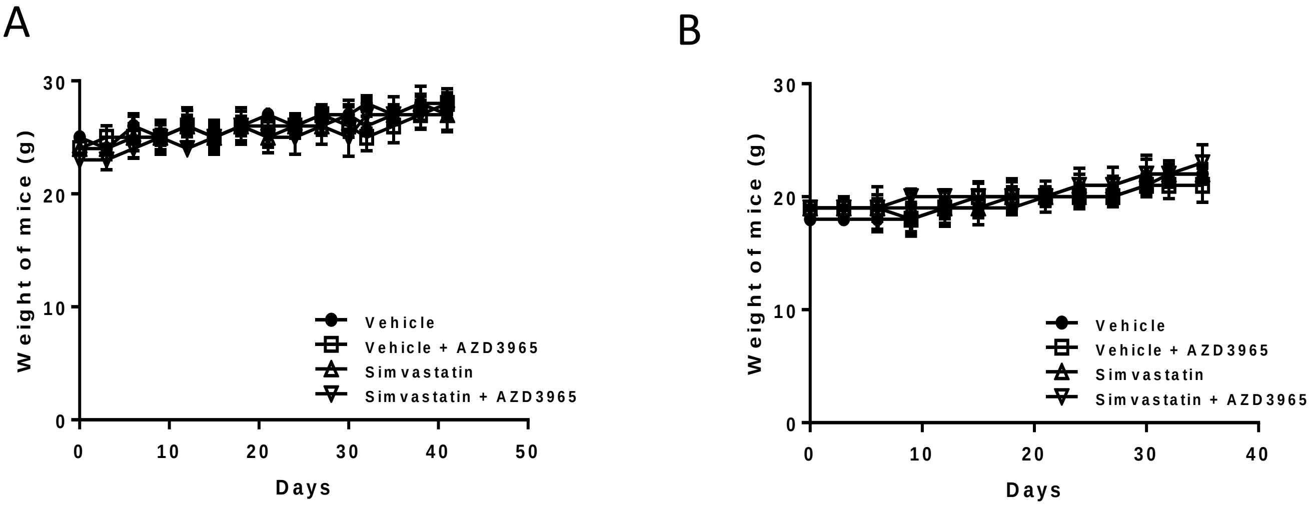

Supplementary Figure 4: Body weights of tumour-bearing mice were not affected by treatment. Athymic CD-1 Nude mice received s.c. injections of FaDu cells (A) and SCID mice received s.c. injections of CaL-27 cells (B). Body weights were measured every 2–3 d during the time period of treatment with vehicle, simvastatin (10mg/kg), AZD3965 (10 days), or the combination of simvastatin (10mg/kg) and AZD3965.

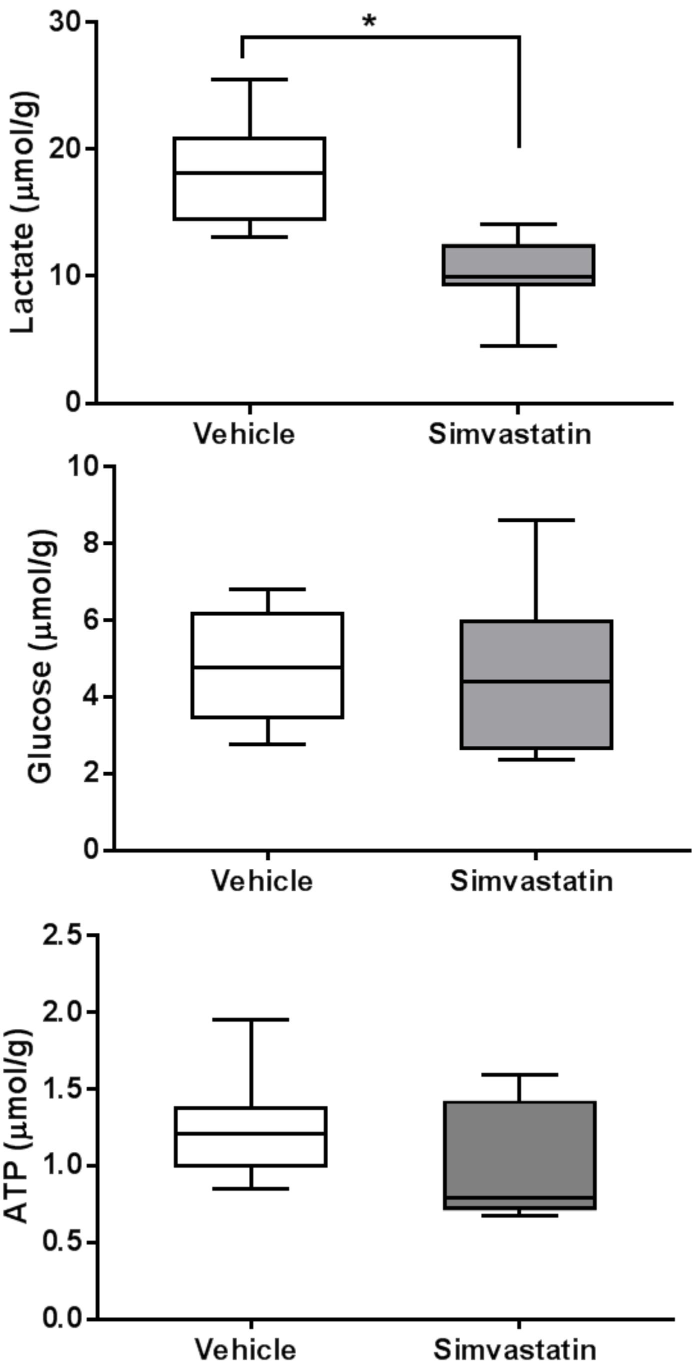

Supplementary Figure 5: Quantification of ATP, lactate and glucose levels in CaL-27 tumour xenografts by imBI. The data correspond to the mean of at least four independent experiments in which one mouse was used per experimental condition. *$P<0.05$, *t*-test.

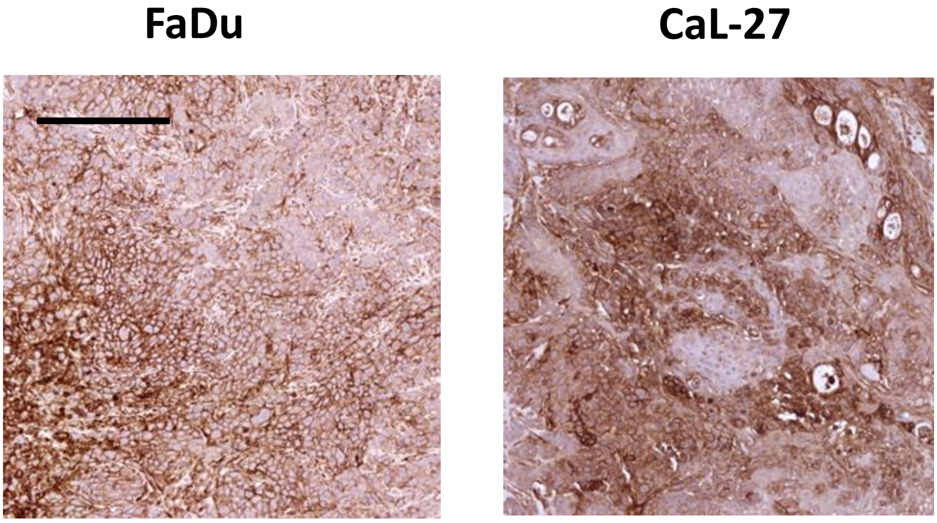

Supplementary Figure 6: Representative images of MCT4 immunohistochemical staining in FaDu and CaL-27 tumours, scale bar represents 200 μm

Characteristics	Patients *n* (%)
Gender	
Male	87 (73)
Female	32 (27)
Age	
Median (Range)	57 (36-90)
Smoking history	
Positive	100 (84)
Negative	17 (14.3)
Unknown	2 (1.7)
Alcohol use	
None	10 (8.4)
Low	41 (34.5)
Moderate	11 (9.2)
High	53 (44.5)
Unknown	4 (3.4)
Clinical Stage	
I	6 (5)
II	19 (16)
III	25 (21)
IV	69 (58)
Statin use	
Positive	19 (16)
Negative	100 (84)

Supplementary Table 2: Patient characteristics at diagnosis

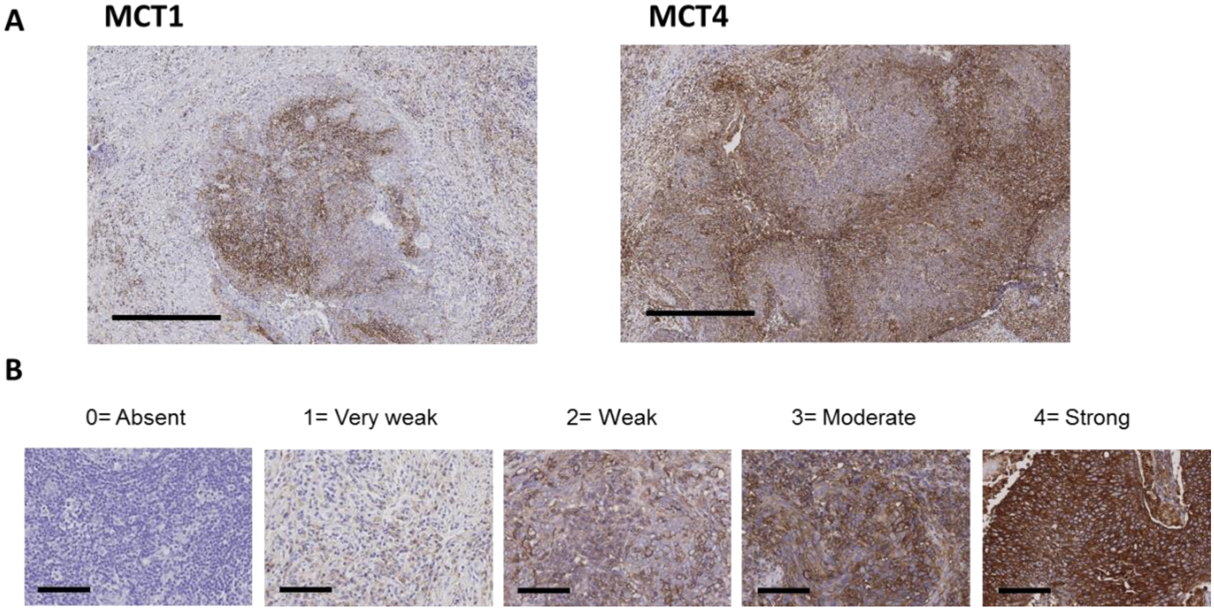

Supplemental Figure 7: A, Representative images of MCT1 and MCT4 staining in HNSCC clinical biopsies. Scale bar represents 500 µm. B, Staining intensities used in scoring IHC sections stained with MCT1 and MCT4 antibodies. Scale bar represents 100 µm.
